# Selected occupational characteristics and change in leukocyte telomere length over 10 years: The Multi-Ethnic Study of Atherosclerosis (MESA)

**DOI:** 10.1371/journal.pone.0204704

**Published:** 2018-09-27

**Authors:** Kaori Fujishiro, Belinda L. Needham, Paul A. Landsbergis, Teresa Seeman, Nancy Swords Jenny, Ana V. Diez Roux

**Affiliations:** 1 Division of Surveillance, Hazard Evaluations, and Field Studies, National Institute for Occupational Safety and Health, Cincinnati, Ohio, United States of America; 2 Department of Epidemiology and Center for Social Epidemiology and Population Health, University of Michigan, Ann Arbor, Michigan, United States of America; 3 Department of Environmental and Occupational Health Sciences, State University of New York Downstate Medical Center, Brooklyn, New York, United States of America; 4 Department of Medicine, University of California Los Angeles, Los Angeles, California, United States of America; 5 Department of Pathology, University of Vermont, Burlington, Vermont, United States of America; 6 Department of Epidemiology and Biostatistics, Dornsife School of Public Health, Drexel University, Philadelphia, Pennsylvania, United States of America; Tokyo Daigaku, JAPAN

## Abstract

Telomere length (TL) is considered as a marker of cell senescence, but factors influencing the rate of TL attrition are not well understood. While one previous study reported the association of occupation and TL, many subsequent studies have failed to find the association. This may be due to heterogeneity within the samples and cross-sectional designs. This longitudinal study examines two occupational characteristics, occupational complexity and hazardous conditions, as predictors of TL attrition in gender- and race/ethnicity-stratified analysis. Leukocyte TL (expressed as T/S ratio) was measured twice over a 10-year period in a multi-racial sample (n = 914). Linear mixed effect models were used to estimate TL attrition associated with occupational complexity and hazardous conditions. Analysis was stratified by gender and race/ethnicity (white, African American, and Latino) and controlled for baseline age, baseline TL, and time since baseline. Higher occupational complexity was associated with slower rates of TL attrition only among white men. Hazardous conditions were not associated with TL attrition for any gender-and-race/ethnicity stratified group. Occupational complexity may influence TL attrition, but the different findings for white men and other groups suggest that a more comprehensive framework is needed to better understand the potential link between occupational characteristics and biological aging.

## Introduction

The length of telomeres (TL), the DNA sequences at the ends of chromosomes, becomes shorter through repeated mitosis. TL is thus considered as a biomarker of aging, and shorter TL has been associated with various age-related illnesses[1] [2]. In 2006, Cherkas et al.[3] reported shorter TL for manual workers compared with non-manual workers. The study sparked considerable interest in socioeconomic status (SES) and biological aging measured by TL. Subsequent studies focusing on various aspects of SES, however, have not reported consistent findings. Occupation, the aspect of SES used in the Cherkas et al. study, was repeatedly found not associated with TL [4–8].

Yet, it is worth revisiting the question of occupation as an SES aspect potentially influencing biological aging for several reasons. First, all previous studies on occupation and TL, including the Cherkas study, are cross-sectional and therefore do not allow inference regarding the rate of TL attrition, which has been suggested as a more sensitive predictor of morbidity and mortality than a single measurement of TL [9, 10]. Also, the occupational categorization used in a US study[5] may not have captured the job characteristics crucial to telomere attrition. In predicting a wide range of health status, job control has been the most consistent [11]. However, the US Census-based occupational categories (e.g., management, professional, service) do not capture this important aspect. For example, aircraft pilots and parking lot attendants are both under “transportation occupations.” Braveman et al.[12] have declared that the US Census categories are “not intended—and do not appear to be meaningful—as SES measures” (p. 2883). More specific characteristics of work, such as job control and hazardous working conditions, may be more appropriate in investigating the impact of work on TL attrition [13].

The occupational categories used in European studies as SES indicators reflect the skill and power aspects of work; however, many studies still did not find a significant association between occupation and TL. A possible reason, besides being cross-sectional, may be heterogeneity of samples. Cherkas et al.[3] used a female only sample whereas many studies with null findings[4–8] analyzed both genders together. Women’s occupational experience can vary more widely than men’s[14] because of social contexts, such as marriage, reproductive circumstances, the spouse’s career change, and family caregiving [14, 15]. Moreover, longitudinal studies reported that women have longer TL at any point of measurement and also had slower rates of TL attrition [16, 17], especially in older age [16]. That is, gender differences exist not only in occupational exposures but also in TL attrition as well. Then, if both genders were analyzed together, such confounding could affect results.

A similar case can be made for separate analyses by race/ethnicity. African Americans and Latinos are not only overrepresented in less complex and more hazardous jobs [18], but also more likely to have fewer opportunities to accumulate skills on the job during their working years [19]. That is, the experience of work even in the same job could be different between whites and other racial/ethnic groups. In addition, current knowledge on TL and race/ethnicity is unclear. One cross-sectional study reported that African Americans had on average a longer TL than whites [20]. Some longitudinal studies have shown that African Americans have a faster rate of TL attrition [21], but this could be because in these studies African Americans had longer TL at baseline [20]. Because of the racially patterned experience of work, analyzing multiple racial/ethnic groups together, even with statistical control, would obscure the potential impact of occupation on TL attrition.

This study examines the association of two job characteristics with TL attrition over ten years in gender- and race/ethnicity-stratified analyses. We specifically focus on occupational complexity [22], a similar but broader construct than job control [23], and hazardous working conditions (i.e., exposure to toxins, harsh weather, noise, extreme temperatures). As Fujishiro et al.[13] argue, these are tangible “manifestations of socioeconomic position that cannot be captured by education or income, or adequately approximated by occupational categories” (p. 498). We hypothesized that higher occupational complexity would be associated with slower rates of TL attrition whereas more hazardous working conditions would be associated with faster rates.

## Methods

### Participants and data collection

The data for this study were collected from a subsample of the Multi-Ethnic Study of Atherosclerosis (MESA), a cohort study designed to investigate the etiology of cardiovascular disease (CVD) [24]. Over 6800 community-dwelling adults, between 45 and 84 years of age and without clinical CVD, participated in MESA. Of those, 1295 were randomly selected for an ancillary study, MESA Stress. Blood samples were collected in MESA Exam 1 (baseline, 2000–2002) and Exam 5 (follow-up, 2010–2011) and analyzed for telomere length. The MESA Stress study protocol was approved by the Institutional Review Boards (IRBs) of the National Heart, Lung, and Blood Institute, Columbia University, University of California Los Angeles, and Johns Hopkins University. This analysis was approved by the IRB of the National Institute for Occupational Safety and Health (NIOSH). All participants provided written informed consent.

Of the 1295 MESA Stress participants, 97 were removed because they had only one TL measurement. Those who never worked outside home (n = 42) and additional 19 with insufficient information on occupation were excluded. Of the remaining 1137, 223 held the job <10 years, and for 113 participants we do not have information on the duration of their job. Because we expect the impact of occupational characteristics to be long term, the 223 with <10 years on the job were excluded. We kept the 113 with unknown job tenure, but ran sensitivity analyses without them. Including the 113, the analytic sample contained 914 participants. Compared to the 914, those excluded were on average younger (56.1 years old vs. 62.0 years old, p < .0001) and correspondingly had longer TL at the baseline (0.95 vs. 0.91, p < .01). However, the average years of follow up was similar (9.50 years vs. 9.45 years, p = 0.11) and so was the change in TL over 10 years (-0.217 vs. -0.220, p = 0.78).

### Measures

#### Leukocyte telomere length

DNA from baseline and follow-up exams was isolated and underwent a standardized procedure at the Collaborative Studies Clinical Laboratory at University of Minnesota Medical Center, Fairview (Minneapolis, MN) from packed EDTA and citrate cells that were frozen at -70C. The DNA extraction and purification method used sodium dodecylsulfate cell lysis followed by a salt precipitation method for protein removal. A mean yield of 39.1 μg DNA/mL packed cell was obtained, and DNA was of high quality (mean purity A260/280 = 1.77) and high molecular weight as determined by gel electrophoresis.

The telomere length assays were performed in the laboratory of Dr. Elizabeth Blackburn at the University of California, San Francisco, using the quantitative polymerase chain reaction (PCR) method to measure telomere length relative to the single-copy gene (human beta-globulin) (T/S ratio), as described in detail elsewhere.[25, 26] Each sample was assayed 3 times on duplicate wells, resulting in 6 data points. Sample plates were assayed in groups of three plates, and no two plates were grouped together more than once. Each assay plate contained 96 control wells, which included 8 standard curves using 3-fold serial dilution of 8 control DNA samples from various cancer cell lines for normalizing batch to batch variations. Any assay run with 8 or more invalid control wells was considered a failed run and was excluded from further analysis (100% of runs passed this criterion). The mean of the T/S values was calculated, and the largest or the smallest T/S value in the set (whichever deviated most from the mean) was marked as a potential outlier. Then the mean of the T/S value was calculated without the potential outlier. If the absolute value of the log of the ratio between the recalculated mean (excluding the potential outlier) to the value of the potential outlier was greater than 0.4, then the value was marked as an outlier (99.8% of all samples contained no outliers). DNA samples were coded and the lab was blinded to all other measurements in the study. The average inter-assay coefficient of variation was 2.9% ± 2.1%.

#### Occupational characteristics

Each participant reported one job at baseline by answering four open-ended questions (e.g., job title, main duties). The participants’ description of the job was coded by trained coders at NIOSH with the Census 2002 Occupation Codes, which were used as the link to the Occupational Information Network (O*NET) database. O*NET is a comprehensive database of job characteristics increasingly used as a job exposure matrix.[27] We used the occupational characteristics of the job reported at baseline and treated them as time-invariant variables. In this cohort of 914 older adults, 471 (51.5%) were no longer in the workforce and reported the main job in life; the others who were still working reported their current job. This could be the main job in life or a post-retirement job for supplemental income, but we do not have information to distinguish them. To address this ambiguity, we conducted a sensitivity analysis excluding those who were still working at baseline. Of the 443 participants who were still in the workforce, 266 remained in the same job throughout the follow-up duration, 141 retired from the job over the course of the study, and only 36 (3.9% of the sample of 914) changed jobs.

As a measure of occupational complexity, we used *substantive complexity of work*, which has been associated with morbidity and mortality [13, 28]. Following Hadden et al.,[29] we created a measure of substantive complexity of work as the mean score of 11 items from O*NET such as inductive and deductive reasoning, information synthesis, critical thinking, and decision making. Cronbach’s alpha for the 11 items was 0.96. Because the item scores do not have intrinsic meaning (i.e., no value over which the level of substantive complexity is health protective), we standardized the score with the mean of zero and the standard deviation of one in the O*NET database (i.e., the score of 0 means that the job has the average level of complexity among all jobs in the US labor market). Higher scores indicate higher levels of occupational complexity.

Hazardous working conditions was represented with the mean score of 8 O*NET items such as exposures to destructive noise, extreme temperatures, high places, outdoors exposed to all weather conditions. Cronbach’s alpha for the 8 items was 0.95. We standardized the score with the mean of zero and the standard deviation of one, with higher scores indicating more hazardous conditions expected in the job. Examples of job titles with high and low scores on these two characteristics can be found in S1 and S2 Tables.

#### Demographic characteristics

Age in years, gender, race/ethnicity (non-Hispanic white, African American, or Hispanic/Latino) were asked on the self-administered questionnaire at the baseline. To indicate the level of education completed, the participants had nine response options, ranging from “no schooling (= 0)” to “Graduate or professional school (= 8).” Education was treated as a continuous variable.

### Statistical analysis

All analyses were stratified by race/ethnicity and gender. After describing sample characteristics, we estimated the association of the occupational characteristics and the rate of TL attrition by including the interaction term between time since baseline and each of the occupational characteristics (examined separately). Coefficients for these interaction terms represent the association of occupational characteristics with the change in TL. We used “hybrid models” that estimate the fixed effects of time and other person-level time-invariant characteristics while also estimating the individual-level random intercept to account for the repeated nature of the measurements of TL [30]. Hybrid models allow us to control for both measured and unmeasured time-invariant characteristics in estimating time trends. The only time-varying covariate in our analyses was time since baseline, which was centered to person-level means. Robust standard errors were estimated.

With each of the race/ethnicity-and-gender stratified samples, we first modeled 10-year TL attrition as a function of only time and the interaction between time and the job characteristic (Model 1). Then we included the interactions between time and baseline TL as well as baseline age (Model 2) based on findings from longitudinal studies that the rate of TL attrition is highly dependent on baseline TL (i.e., the longer the TL, the more rapid the attrition is)[31, 32] and age.[16, 17] Finally, we included the interaction between time and education (Model 3). Although the association between education and TL has been inconsistent [3, 8], one study reported a significant association for whites [20]. Because education largely determines types of jobs available, it may confound the associations of occupational characteristics with TL. See S1 File for model specification.

## Results

The sample characteristics are presented in Table 1 by gender and race/ethnicity. The average age was similar across the gender-race/ethnicity groups, but whites were slightly older. Despite their higher average age, whites had longer telomeres at baseline. For both gender groups, the average level of occupational complexity was the highest for whites and the lowest for Latinos. The average level of hazardous working conditions was the highest for Latino men and the lowest for white women, but the racial/ethnic difference in women was not as prominent as in men. See S1 and S2 Figs for the mean values of these measures by race and gender. See S3 Fig for individuals’ telomere length change by race and gender.

**Table 1:** Demographic characteristics, baseline TL, and change in TL over 10 years.

	Men			Women		
Characteristic	White	African American	Latino	White	African American	Latino
N	118	122	183	118	163	210
Age at baseline, years, mean (SD)	62.1	61.4	61.5	63.7	61.5	62.1
	(8.9)	(9.1)	(9.9)	(9.8)	(9.5)	(9.2)
Baseline Telomere length, T/S ratio, mean (SD)	0.93	0.85	0.89	0.93	0.92	0.92
	(0.23)	(0.20)	(0.21)	(0.19)	(0.20)	(0.21)
Change in telomere length in 10 years, T/S ratio, mean (SD)	-0.26	-0.17	-0.22	-0.23	-0.20	-0.24
	(0.21)	(0.19)	(0.20)	(0.19)	(0.19)	(0.19)
Occupational complexity, standardized,^1^ mean (SD)	0.84	-0.02	-0.47	0.34	-0.06	-0.60
	(0.80)	(1.03)	(1.10)	(0.94)	(1.00)	(0.99)
Hazardous working conditions, standardized,^1^ mean (SD)	-0.42	-0.07	0.00	-0.82	-0.61	-0.52
	(0.86)	(0.88)	(0.80)	(0.40)	(0.50)	(0.61)
Education, %						
No schooling	0.0	0.0	0.6	0.0	0.0	1.0
Grades 1–8	0.9	0.8	26.2	1.7	1.8	27.6
Grade 9–11	4.2	8.2	14.2	2.5	6.8	13.8
Completed high school/GED	9.3	19.7	20.8	14.4	28.8	24.8
Some college but no degree	15.3	25.4	13.1	23.7	23.9	14.8
Technical school certificate	1.7	10.7	8.2	1.7	9.8	5.7
Associate degree	3.4	4.9	4.9	2.5	5.5	5.2
Bachelor’s degree	23.7	18.0	7.7	22.9	11.7	4.3
Graduate or professional degree	41.5	12.3	4.4	30.5	11.7	2.9

Notes. TL = telomere length; SD = standard deviation.

^1^ mean = 0, standard deviation = 1

Table 2 shows the association between substantive complexity of work and the rate of TL attrition by gender and race/ethnicity. In the models adjusted only for time (Model 1), only white men showed an association approaching statistical significance (p = 0.07) in the expected direction: The higher the level of substantive complexity of work, the slower the rate of TL attrition. When the interactions of time with baseline TL and age were included in the model (model 2), the association for white men became statistically significant, but when the interaction between time and education was added to the model (Model 3), it was no longer significant. In model 3, the interaction between time and education was not significant either (est. = 0.01, SE = 0.01, p = 0.30, see S3 Table for full results), even though without occupational complexity, education was statistically significant for white men (as shown in S3 and S4 Tables). Non-significant coefficients in Model 3 were expected given the high correlation between education and substantive complexity (Spearman’s rho = 0.52, p < .0001 for white men, see S5 Table for other groups). Neither substantive complexity nor education was associated with the rates of TL attrition for any other gender-race/ethnicity group. Table 3 presents the results for hazardous working conditions. This occupational characteristic was not associated with rates of TL attrition in any group.

**Table 2:** Estimated change in 10-year telomere attrition associated with 1-SD increase in occupational complexity by gender and race/ethnicity.

Gender and Race/ethnicity	n	Model 1: adjusted for time only		Model 2: adjusted for time, time x baseline TL, and time x baseline age		Model 3: adjusted for time, time x baseline TL, time x baseline age, and education	
		Est.	95%CI	Est.	95%CI	Est.	95%CI
Men							
White	118	0.05	(-0.00, 0.10)	0.03	(0.00, 0.05)	0.02	(-0.01, 0.05)
African American	122	-0.01	(-0.04, 0.03)	-0.00	(-0.03, 0.02)	0.00	(-0.04, 0.03)
Latino	183	-0.01	(-0.04, 0.02)	-0.00	(-0.02, 0.02)	0.00	(-0.02, 0.02)
Women							
White	118	0.02	(-0.02, 0.06)	0.01	(-0.02, 0.04)	0.01	(-0.02, 0.05)
African American	163	-0.01	(-0.03, 0.03)	-0.01	(-0.03, 0.01)	0.00	(-0.03, 0.02)
Latino	210	-0.02	(-0.04, 0.01)	-0.01	(-0.03, 0.00)	-0.01	(-0.03, 0.00)

Notes. The estimate is the regression coefficient for the interaction between follow-up time and occupational complexity. A negative coefficient indicates greater 10-year telomere attrition. Follow-up time was centered to the individual’s average time. Exam 1 telomere length and age were centered to the population mean. SD = standard deviation; Est. = estimate; CI = confidence interval; TL = telomere length.

**Table 3:** Estimated change in 10-year telomere attrition associated with 1-SD increase in hazardous working conditions by gender and race/ethnicity.

Gender and Race/ethnicity	n	Model 1: adjusted for time only		Model 2: adjusted for time, time x baseline TL, and time x baseline age		Model 3: adjusted for time, time x baseline TL, time x baseline age, and education	
		Est.	95%CI	Est.	95%CI	Est.	95%CI
Men							
White	118	-0.01	(-0.06, 0.03)	-0.01	(-0.04, 0.01)	-0.01	(-0.03, 0.02)
African American	122	0.01	(-0.03, 0.05)	0.01	(-0.02, 0.04)	0.01	(-0.02, 0.04)
Latino	183	-0.03	(-0.07, 0.00)	-0.02	(-0.04, 0.01)	-0.02	(-0.04, 0.01)
Women							
White	118	-0.00	(-0.10, 0.10)	0.04	(-0.01, 0.10)	0.05	(-0.02, 0.10)
African American	163	-0.05	(-0.11, 0.01)	-0.02	(-0.05, 0.02)	-0.01	(-0.05, 0.02)
Latino	210	0.00	(-0.04, 0.05)	0.01	(-0.02, 0.03)	0.00	(-0.02, 0.03)

Notes. The estimate is the regression coefficient for the interaction between follow-up time and hazardous working conditions. A negative coefficient indicates greater 10-year telomere attrition. Follow-up time was centered to the individual’s average time. Exam 1 telomere length and age were centered to the population mean. SD = standard deviation; Est. = estimate; CI = confidence interval; TL = telomere length.

As a sensitivity analysis, we removed those who potentially had less than 10 years of exposure to the occupational characteristics. The results were similar. Next, we restricted the analysis to those who were no longer in the workforce at baseline (i.e., the reported job was the main job in life) so as to remove those who might have reported a post-retirement, short-term job. The results were again similar. The analysis of those who were still working at baseline did not show any significant associations. Treating occupational characteristics as time-varying variables did not show any associations. These results suggest that if occupational characteristics were associated with the rate of TL attrition, it would not be particularly acute. Lastly, as a comparison, instead of stratifying the sample, we used the entire sample and controlled for race and gender. The occupational characteristics were not associated with TL attrition (full regression results are shown in S6 and S7 Tables).

## Discussion

This longitudinal study examined the association of occupational characteristics with the rate of TL attrition in a gender and race/ethnicity stratified sample of older adults. The results were largely null; but among white men, occupational complexity was associated with slower rates of TL attrition, and this association was clearer when baseline TL and age were controlled for. The rate of TL attrition is highly dependent on baseline TL [31, 32] and baseline age [16, 17], and TL is also reported to be highly heritable [33]. Controlling for the effect of baseline TL is, therefore, to remove the potentially sizable heritability in TL along with potential impacts of previous life experiences on TL. Among white men, we found that after such adjustments, the association between occupational complexity and slower rates of TL attrition, even though the magnitude of the association was smaller, became more highly statistically significant. This suggests that occupational complexity may influence TL attrition rates, for white men, independent from heritability or previous life experiences.

In our data, the pattern of the association between occupational complexity and TL attrition was similar to the association between education and TL attrition: that is, only among white men, education was associated with TL attrition (S4 Table). However, when both were in the model, neither education nor the occupational characteristics had a significant association with the rate of TL attrition. Because education is highly correlated with occupation (S5 Table), it is not possible to separate their association with TL attrition, but occupational complexity may be part of the mechanism through which education affects TL attrition. Because education has not been consistently associated with TL [20] or the rate of TL attrition [32], it is difficult to draw a conclusion at this point.

The association of highly complex jobs and slower rates of TL attrition was not observed African American or Latino men or women of any race/ethnicity. There are several possibilities for the null findings. First, the true association may be small in magnitude and could only be observed in white men because a larger proportion of them had highly complex jobs. Second, the highly complex jobs that African American men, Latino men, and women of all races/ethnicities have may also come with other aspects of work that are detrimental to biological aging. When members of the underrepresented groups achieve a higher power position in the workplace, which is likely to be more complex, they may be under scrutiny, perceived or actual, to perform better than their white counterparts [34]. Negative aspects of highly complex jobs for women as well as African American and Latino men may have negated any benefit from such jobs. Also, although education and occupational complexity were positively correlated in all gender-race/ethnicity groups, white men at all education levels had more complex jobs, consistent with the well-documented “diminished returns” of education for women as well as African American and Latino men [35]. There may be a threshold level for substantive complexity to be beneficial.

As for women, the null findings in this study may be a result of intricate relationships between work and other aspects of life [14]. Because women’s workforce participation and work experience are determined by many factors, not incorporating caregiving obligations and support from the partner, for example, may have obscured the association between the two occupational characteristics and TL attrition. A related issue to be considered is the work of homemakers. There is no standardized way to capture the complexity or hazard of homemaking, which is the reason for excluding those who did not report work outside home from this study. This exclusion disproportionally affected Latinas in our sample (i.e., 34 Latinas were excluded; while 0 to 3 were excluded from other race/gender groups). A more comprehensive framework for analyzing women’s health and the role of work, including homemaking, with explicit attention to family and social contexts, as Artazcoz et al.[14] propose, is an imperative.

### Strengths and limitations

This study adds to the still small number of longitudinal studies analyzing TL attrition in a population-based sample. The diversity in race/ethnicity and occupations in our sample is a strength. The use of O*NET allows us to analyze job characteristics important for health in general. A major limitation is the lack of job tenure information for many participants. Given the older cohort, we assume that at least those who were already retired at baseline had kept the same job for a substantial duration of time [36]. However, because white men tend to stay in the workforce more continuously than non-white men or women of any racial/ethnic background [37], there may have been systematic differences in exposure duration by race and gender. This study examined only two occupational characteristics and analyzed them separately. Although these are important aspects of work [13], other occupational characteristics such as the type of employment (e.g., full-time employee vs. temporary or subcontract employees) may have impact on TL attrition. As for the TL measurements, because we do not have data on cell compositions, the observed results may be attributable to differences in cell compositions at baseline and follow-up. Also, we used the average TL across all leukocyte cell types. Because TL in different cell types within the same individual could vary [38], more uniform cell populations may have provided a clearer result.

### Conclusions

In this longitudinal study, expected associations between occupational complexity and the rate of TL attrition were found only among white men. If high occupational complexity indeed protects against TL attrition, other factors—most likely both social structural as well as family and individual-level factors—may hinder women and minority men from experiencing the benefits. Exploring these factors will help understand the racial/ethnic inequalities in biological aging.

## Supporting information

S1 Table Job titles with high and low scores on substantive complexity and hazardous working conditions for men by race/ethnicity. (DOCX)

S2 Table Job titles with high and low scores on occupational complexity and hazardous working conditions for women by race/ethnicity. (DOCX)

S3 Table Estimated change in 10-year telomere attrition by occupational complexity and education among white men from the linear mixed effects (hybrid) model with a random intercept and robust standard errors. (DOCX)

S4 Table Estimated change in 10-year telomere attrition associated with a 1-unit increase in education by gender and race/ethnicity. (DOCX)

S5 Table Spearman’s rho (p-value) between education and each of the two occupational characteristics. (DOCX)

S6 Table Estimated change in 10-year telomere attrition by occupational complexity and sociodemographic characteristics from the linear mixed effects (hybrid) model with a random intercept and robust standard errors (n = 914). (DOCX)

S7 Table Estimated change in 10-year telomere attrition by hazardous working conditions and sociodemographic characteristics from the linear mixed effects (hybrid) model with a random intercept and robust standard errors (n = 914). (DOCX)

S1 Fig Least square mean and standard error of the substantive complexity of work score (standardized) by gender and race/ethnicity. (DOCX)

S2 Fig Least square mean and standard error of the hazardous working conditions score (standardized) by gender and race/ethnicity. (DOCX)

S3 Fig Individual trajectories of telomere length between baseline and follow-up by gender and race (spaghetti plots). (DOCX)

S1 File Model specification. (DOCX)
