# Supplementary material for: Selected occupational characteristics and change in leukocyte telomere length over 10 years: The Multi-Ethnic Study of Atherosclerosis (MESA)
Source: PLoS One. 2018 Sep 27;13(9):e0204704. doi: 10.1371/journal.pone.0204704 (PMC6160145; doi:10.1371/journal.pone.0204704)
Supplement: S1 Table — (DOCX) [file pone.0204704.s001.docx]

S1 Table. Job titles with high and low scores on substantive complexity and hazardous working conditions for men by race/ethnicity

|  | White men | | Black men | | Latino men | |
| --- | --- | --- | --- | --- | --- | --- |
| Occupational Characteristic | Job title | O*NET score^1^ | Job title | O*NET score^1^ | Job title | O*NET score^1^ |
| Substantive complexity  Highest (protective) | Judges and magistrates | 2.29 | Veterinarians | 1.95 | Chief executives | 1.92 |
|  | Dentists | 2.25 | Medical scientists | 1.92 | Financial managers | 1.45 |
|  | Lawyers | 2.09 | Education administrators | 1.58 | Engineering managers | 1.33 |
|  | Astronomers and physicists | 1.97 | Legislators | 1.49 | Industrial engineers | 1.28 |
|  | Veterinarians | 1.95 | Financial managers | 1.45 | Mechanical engineers | 1.22 |
|  | **…** |  | **…** |  | **…** |  |
| Lowest (harmful) | Nursing and home health aides | -0.75 | Helpers--production workers | -1.71 | Grounds maintenance workers | -1.94 |
|  | Driver/sales workers and truck drivers | -0.76 | Postal service mail carriers | -1.74 | Packers and packagers, hand | -1.94 |
|  | Models, demonstrators, and product promoters | -0.89 | Janitors and building cleaners | -1.78 | Laundry and dry-cleaning workers | -2.04 |
|  | Office clerks, general | -0.91 | Maids and housekeeping cleaners | -1.82 | Pressers, textile, garment | -2.16 |
|  | Painters, construction and maintenance | -1.11 | Food preparation workers | -2.61 | Food preparation workers | -2.61 |
|  |  |  |  |  |  |  |
| **Hazardous working conditions**  Lowest (protective)  Highest (harmful) | Personal financial advisors | -1.57 | Insurance sales agents | -1.28 | Personal financial advisors | -1.57 |
|  | Chiropractors | -1.50 | Word processors and typists | -1.27 | Receptionists and information clerks | -1.32 |
|  | Financial analysts | -1.37 | Human resources, training, and labor relations specialists | -1.27 | Sales and related workers, all other | -1.28 |
|  | Bookkeeping, accounting, and auditing clerks | -1.32 | Human resources managers | -1.24 | Insurance sales agents | -1.28 |
|  | Computer software engineers | -1.31 | Financial managers | -1.23 | File clerks | -1.28 |
|  | **…** |  | **…** |  | **…** |  |
|  | Fire fighters | 1.56 | Control and valve installers and repairers | 1.66 | Maintenance and repair workers, general | 1.47 |
|  | First-line supervisors/managers of fire fighting and prevention workers | 1.57 | Electricians | 1.69 | Construction laborers | 1.49 |
|  | Heavy vehicle and mobile equipment service technicians and mechanics | 1.90 | Roofers | 1.85 | Carpenters | 1.50 |
|  | Heating, air conditioning, and refrigeration mechanics and installers | 1.95 | Stationary engineers and boiler operators | 1.99 | Electricians | 1.69 |
|  | Stationary engineers and boiler operators | 1.99 | Miscellaneous plant and system operators | 2.06 | Elevator installers and repairers | 1.99 |

^1^ mean=0, standard deviation=1
