# Supplementary material for: Selected occupational characteristics and change in leukocyte telomere length over 10 years: The Multi-Ethnic Study of Atherosclerosis (MESA)
Source: PLoS One. 2018 Sep 27;13(9):e0204704. doi: 10.1371/journal.pone.0204704 (PMC6160145; doi:10.1371/journal.pone.0204704)
Supplement: S2 Table — (DOCX) [file pone.0204704.s002.docx]

Table S2. Job titles with high and low scores on occupational complexity and hazardous working conditions for women by race/ethnicity

|  | White women | | Black women | | Latino women | |
| --- | --- | --- | --- | --- | --- | --- |
| Occupational Characteristic | Job title | O*NET standardized score^1^ | Job title | O*NET standardized score^1^ | Job title | O*NET standardized score^1^ |
| Substantive complexity  Highest (protective) | Physicians and surgeons | 2.31 | Chief executives | 1.92 | Dentists | 2.25 |
|  | Lawyers | 2.09 | Registered nurses | 1.59 | Registered nurses | 1.59 |
|  | Chief executives | 1.92 | Education administrators | 1.58 | Physician assistants | 1.36 |
|  | Registered nurses | 1.59 | Biological scientists | 1.54 | Other healthcare practitioners and technical occupations | 1.23 |
|  | Education administrators | 1.58 | Dietitians and nutritionists | 1.37 | Human resources managers | 1.18 |
|  | **…** |  | **…** |  | **…** |  |
| Lowest (harmful) | Telephone operators | -1.02 | File clerks | -1.57 | Maids and housekeeping cleaners | -1.82 |
|  | Postal service clerks | -1.14 | Cashiers | -1.59 | Packers and packagers, hand | -1.94 |
|  | Cashiers | -1.59 | Janitors and building cleaners | -1.78 | Laundry and dry-cleaning workers | -2.04 |
|  | Sewing machine operators | -1.62 | Maids and housekeeping cleaners | -1.82 | Pressers, textile, garment, and related materials | -2.16 |
|  | Food preparation workers | -2.61 | Food preparation workers | -2.61 | Food preparation workers | -2.61 |
|  |  |  |  |  |  |  |
| **Hazardous working conditions**  Lowest (protective)  Highest (harmful) | Tax preparers | -1.50 | Receptionists and information clerks | -1.32 | Personal financial advisors | -1.57 |
|  | Statisticians | -1.48 | Bookkeeping, accounting, and auditing clerks | -1.32 | Travel agents | -1.45 |
|  | Accounts clerks | -1.46 | Sales and related workers, all other | -1.28 | Loan interviewers and clerks | -1.38 |
|  | Travel agents | -1.45 | File clerks | -1.28 | Receptionists and information clerks | -1.32 |
|  | Receptionists and information clerks | -1.32 | Word processors and typists | -1.27 | Telemarketers | -1.25 |
|  | **…** |  | **…** |  | **…** |  |
|  | Athletes, coaches, umpires, and related workers | 0.11 | Driver/sales workers and truck drivers | 0.74 | Crossing guards | 0.89 |
|  | Printing machine operators | 0.13 | Rolling machine setters, operators, and tenders, metal and plastic | 0.96 | Painting workers | 0.95 |
|  | Transportation attendants | 0.13 | Supervisors, protective service workers, all other | 1.05 | Production workers, all other | 1.36 |
|  | Other life, physical, and social science technicians | 0.20 | Police and sheriff's patrol officers | 1.15 | Aircraft mechanics and service technicians | 1.56 |
|  | Inspectors, testers, sorters, samplers, and weighers | 0.41 | Emergency medical technicians and paramedics | 1.73 | Structural iron and steel workers | 2.33 |

^1^ mean=0, standard deviation=1
