# Supplementary material for: Selected occupational characteristics and change in leukocyte telomere length over 10 years: The Multi-Ethnic Study of Atherosclerosis (MESA)
Source: PLoS One. 2018 Sep 27;13(9):e0204704. doi: 10.1371/journal.pone.0204704 (PMC6160145; doi:10.1371/journal.pone.0204704)
Supplement: S3 Table — (DOCX) [file pone.0204704.s003.docx]

| Table S3. Estimated change in 10-year telomere attrition by occupational complexity and education among white men from the linear mixed effects (hybrid) model with a random intercept and robust standard errors | | | | | | | | | |
| --- | --- | --- | --- | --- | --- | --- | --- | --- | --- |
| Variable | Est. | 95%CI | Est. | 95%CI | Est. | 95%CI | Est. | 95%CI |  |
| Intercept | 0.81 | (0.78, 0.83) | 0.81 | (0.78, 0.83) | 0.81 | (0.78, 0.83) | 0.81 | (0.78, 0.83) |  |
| Time | -0.30 | (-0.37, -0.24) | -0.30 | (-0.36, -0.24) | -0.31 | (-0.38, -0.24) | -0.27 | (-0.30, -0.23) |  |
| **Time x Complexity** | **0.05** | **(0.00, 0.10)** | -- | -- | **0.03** | **(-0.03, 0.09)** | **0.02** | **(-0.01, 0.05)** |  |
| **Time x Education** | -- | -- | **0.02** | **(0.00, 0.04)** | **0.01** | **(-0.01, 0.03)** | **0.01** | **(-0.01, 0.02)** |  |
| Time x Exam 1 TL | -- | -- | -- | -- | -- | -- | -0.76 | (-0.86, -0.66) |  |
| Time x Exam 1 age | -- | -- | -- | -- | -- | -- | -0.02 | (-0.05, 0.00) |  |
| Notes: Follow-up time was centered to the individual’s average follow-up time and is presented on a 10-year scale. Education (9 levels, treated continuous) and Exam 1 telomere length were centered to the population mean. A negative coefficient for an interaction with time indicates greater 10-year telomere attrition. Est.=estimate; CI=confidence interval; TL=telomere length. | | | | | | | | | |
