# Supplementary material for: Selected occupational characteristics and change in leukocyte telomere length over 10 years: The Multi-Ethnic Study of Atherosclerosis (MESA)
Source: PLoS One. 2018 Sep 27;13(9):e0204704. doi: 10.1371/journal.pone.0204704 (PMC6160145; doi:10.1371/journal.pone.0204704)
Supplement: S4 Table — (DOCX) [file pone.0204704.s004.docx]

Table S4: Estimated change in 10-year telomere attrition associated with a 1-unit increase in education by gender and race/ethnicity

|  |  | Model 1: adjusted for time only | | Model 2: adjusted for time, time x baseline TL, and time x baseline age | | Model 3: adjusted for time, time x baseline TL, time x baseline age, and occupational complexity | |
| --- | --- | --- | --- | --- | --- | --- | --- |
| Gender and Race/ethnicity | n | Est. | 95%CI | Est. | 95%CI | Est. | 95%CI |
| Men |  |  |  |  |  |  |  |
| White | 118 | **0.02** | (0.00, 0.04) | **0.01** | (0.00, 0.02) | 0.01 | (-0.01, 0.02) |
| African American | 122 | 0.00 | (-0.02, 0.02) | 0.00 | (-0.01, 0.01) | 0.00 | (-0.02, 0.02) |
| Hispanic | 183 | 0.01 | (-0.00, 0.02) | 0.00 | (-0.01, 0.01) | 0.00 | (-0.01, 0.01) |
| Women |  |  |  |  |  |  |  |
| White | 118 | 0.01 | (-0.01, 0.02) | 0.00 | (-0.01, 0.01) | 0.00 | (-0.02, 0.01) |
| African American | 163 | -0.01 | (-0.03, 0.00) | 0.00 | (-0.01, 0.01) | 0.00 | (-0.02, 0.01) |
| Hispanic | 210 | 0.00 | (-0.01, 0.01) | 0.00 | (-0.01, 0.01) | 0.00 | (-0.01, 0.01) |

Notes. The estimate is the regression coefficient for the interaction between follow-up time and education (range: 0 (no schooling) to 8 (graduate or professional degree)). A negative coefficient indicates greater 10-year telomere attrition. Follow-up time was centered to the individual’s average time. Exam 1 telomere length and age were centered to the population mean. SD=standard deviation; Est.=estimate; CI=confidence interval; TL=telomere length.
