# Supplementary material for: Selected occupational characteristics and change in leukocyte telomere length over 10 years: The Multi-Ethnic Study of Atherosclerosis (MESA)
Source: PLoS One. 2018 Sep 27;13(9):e0204704. doi: 10.1371/journal.pone.0204704 (PMC6160145; doi:10.1371/journal.pone.0204704)
Supplement: S5 Table — (DOCX) [file pone.0204704.s005.docx]

Table S5: Spearman’s rho (p-value) between education and each of the two occupational characteristics

|  | Correlation coefficient between education and … | |
| --- | --- | --- |
| Gender and Race/ethnicity | Substantive complexity of work (p-value) | Hazardous working conditions (p-value) |
| Men |  |  |
| White | 0.52 (<.*0001*) | -0.28 (<.*01*) |
| African American | 0.36 (<.*0001*) | -0.32 (<.*01*) |
| Hispanic | 0.51 (<.*0001*) | -0.22 (<.*01*) |
| Women |  |  |
| White | 0.54 (<.*0001*) | 0.04 (0.*64*) |
| African American | 0.51 (<.*0001*) | -0.06 (0.*46*) |
| Hispanic | 0.44 (<.*0001*) | -0.28 (<.*0001*) |
