# Supplementary material for: Selected occupational characteristics and change in leukocyte telomere length over 10 years: The Multi-Ethnic Study of Atherosclerosis (MESA)
Source: PLoS One. 2018 Sep 27;13(9):e0204704. doi: 10.1371/journal.pone.0204704 (PMC6160145; doi:10.1371/journal.pone.0204704)
Supplement: S6 Table — (DOCX) [file pone.0204704.s006.docx]

| Table S6. Estimated change in 10-year telomere attrition by occupational complexity and sociodemographic characteristics from the linear mixed effects (hybrid) model with a random intercept and robust standard errors (n=914) | | | | | | | | | | |
| --- | --- | --- | --- | --- | --- | --- | --- | --- | --- | --- |
| Variable | Est. | 95%CI | Est. | 95%CI | Est. | 95%CI | Est. | 95%CI | Est. | 95%CI |
| Intercept | 0.80 | (0.79, 0.81) | 0.80 | (0.79, 0.81) | 0.80 | (0.79, 0.81) | 0.80 | (0.79, 0.81) | 0.80 | (0.79, 0.81) |
| Time | -0.22 | (-0.24, -0.21) | -0.22 | (-0.24, -0.21) | -0.22 | (-0.23, -0.21) | -0.22 | (-0.23, -0.21) | -0.22 | (-0.23, -0.21) |
| **Time x Complexity** | **0.00** | **(-0.02, 0.01)** | **0.00** | **(-0.02, 0.01)** | **0.00** | **(-0.01, 0.01)** | **0.00** | **(-0.02, 0.01)** | **0.00** | **(-0.01, 0.01)** |
| Time x Male | -- | -- | 0.01 | (-0.01, 0.04) | -0.01 | (-0.03, 0.01) | 0.01 | (-0.01, 0.04) | -0.01 | (-0.03, 0.01) |
| Time x Black | -- | -- | 0.05 | (0.02, 0.09) | 0.02 | (0.00, 0.05) | 0.05 | (0.02, 0.09) | 0.02 | (-0.01, 0.04) |
| Time x Hispanic | -- | -- | 0.01 | (-0.02, 0.05) | -0.01 | (-0.03, 0.01) | 0.01 | (-0.02, 0.05) | -0.01 | (-0.04, 0.01) |
| Time x Male x Black | -- | -- | 0.06 | (0.00, 0.13) | 0.02 | (-0.03, 0.06) | 0.06 | (-0.01, 0.13) | 0.02 | (-0.02, 0.07) |
| Time x Male x Hispanic | -- | -- | 0.06 | (-0.01, 0.12) | 0.04 | (0.00, 0.08) | 0.06 | (-0.01, 0.12) | 0.04 | (0.00, 0.08) |
| Time x Exam 1 TL | -- | -- | -- | -- | -0.70 | (-0.74, -0.66) | -- | -- | -0.73 | (-0.78, -0.69) |
| Time x Exam 1 age | -- | -- | -- | -- | -- | -- | 0.01 | (0.00, 0.03) | -0.03 | (-0.04, -0.02) |
| Notes: Follow-up time was centered to the individual’s average follow-up time and is presented on a 10-year scale. Demographic variables and Exam 1 telomere length were centered to the population mean. A negative coefficient for an interaction with time indicates greater 10-year telomere attrition. Est.=estimate; CI=confidence interval; TL=telomere length. | | | | | | | | | | |
