# Supplementary material for: Selected occupational characteristics and change in leukocyte telomere length over 10 years: The Multi-Ethnic Study of Atherosclerosis (MESA)
Source: PLoS One. 2018 Sep 27;13(9):e0204704. doi: 10.1371/journal.pone.0204704 (PMC6160145; doi:10.1371/journal.pone.0204704)
Supplement: S1 Fig — (DOCX) [file pone.0204704.s008.docx]

Figure S1. Least square mean and standard error of the substantive complexity of work score (standardized) by gender and race
