# Supplementary material for: Selected occupational characteristics and change in leukocyte telomere length over 10 years: The Multi-Ethnic Study of Atherosclerosis (MESA)
Source: PLoS One. 2018 Sep 27;13(9):e0204704. doi: 10.1371/journal.pone.0204704 (PMC6160145; doi:10.1371/journal.pone.0204704)
Supplement: S3 Fig — (DOCX) [file pone.0204704.s010.docx]

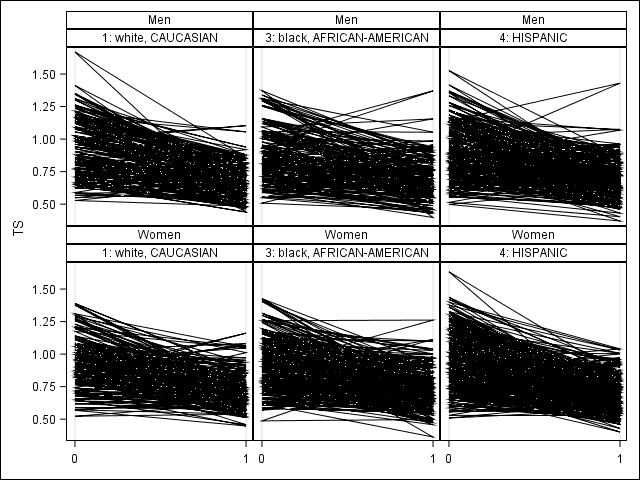


baseline

baseline

baseline

Exam 5

Exam 5

Exam 5

T/S ratio

Figure S3. Individual trajectories of telomere length between baseline and follow-up (Exam 5) by gender and race
