# Supplementary material for: Selected occupational characteristics and change in leukocyte telomere length over 10 years: The Multi-Ethnic Study of Atherosclerosis (MESA)
Source: PLoS One. 2018 Sep 27;13(9):e0204704. doi: 10.1371/journal.pone.0204704 (PMC6160145; doi:10.1371/journal.pone.0204704)
Supplement: S1 File — (DOCX) [file pone.0204704.s011.docx]

The hybrid model specification

$$y_{ij}= \beta_{i0}+\beta_{1}\cdot{Time}_{ij}^{*}+\beta_{2}\cdot{Time}_{ij}^{*}\cdot{Occupation}_{i0}+\beta_{3}\cdot{Time}_{ij}^{*}\cdot{Age}_{i0}+ \beta_{4}\cdot{Time}_{ij}^{*}\cdot{TL}_{i1}+ \beta_{5}\cdot{Time}_{ij}^{*}\cdot{Educaiton}_{i0}+ \epsilon_{ij}$$

$$\beta_{i0}= \beta_{0}+b_{i0}$$

$y_{ij}$: Telomere length for individual *i* at Exam *j*.

${Time}_{ij}^{*}$: Time (years) since baseline exam (Exam 1) for individual *i* at Exam *j.*${Time}_{ij}^{*}$ is centered to individual *i*’s average follow-up time: ${Time}_{ij}^{*}$ = $Time_{ij}- \bar{Time_{i}.}$ where $Time_{ij}$ is the follow-up years and $\bar{Time_{i}.}$ is the average follow-up years for individual *i*.

${Occupation}_{i0}$: The characteristic of individual *i*’s occupation reported at baseline

${Age}_{i0}$: The age of individual *i* reported at baseline

${Educaiton}_{i0}$: The level of education of individual *i* *reported at baseline*

$\beta_{0}$: Model intercept representing the population mean for telomere length over two exams.

$b_{i0}$: Individual-level random intercept representing individual specific deviation from the population mean on telomere length over two exams.

$\beta_{1}$: Average annual change in telomere length in the population.

$\beta_{2}$: Difference in the annual change in telomere lengths associated with a 1-standard-deviation difference in the occupational characteristic.

$\beta_{3}$: Baseline age effect on annual change in telomere length.

$\beta_{4}$: Baseline TL effect on annual change in telomere length.

$\beta_{5}$: Baseline education effect on annual change in telomere length.

$\epsilon_{ij}$: Unexplained random error term.
